# Hepatitis C in healthcare personnel: secondary data analysis of therapies with direct-acting antiviral agents

**DOI:** 10.1186/s12995-018-0197-6

**Published:** 2018-05-25

**Authors:** Claudia Westermann, Dana Wendeler, Albert Nienhaus

**Affiliations:** 1 Competence Centre for Epidemiology and Health Services Research for Healthcare Professionals (CVcare), Institute for Health Services Research in Dermatology and Nursing (IVDP), University Medical Centre Hamburg-Eppendorf (UKE), Martinistr. 52, 20146 Hamburg, Germany; 2 Department of Occupational Medicine, Hazardous Substances and Public Health, Institution for Statutory Accident Insurance and Prevention in the Health and Welfare Services (BGW), Pappelallee 33-37, 22089 Hamburg, Germany

**Keywords:** Hepatitis C virus, Occupational exposure, Blood-borne infections, Healthcare personnel, Direct-acting antiviral agents

## Abstract

**Background:**

Hepatitis C Virus (HCV) infections are blood-borne, generally chronic and are associated with increased morbidity and mortality. The aim of this study is to describe the results of therapies with direct-acting antiviral agents (DAAs) in healthcare personnel.

**Methods:**

Secondary data analysis using data from the Statutory Accident Insurance of the Health and Welfare Service. The study surveyed DAA therapies administered to insured parties (healthcare personnel with an HCV infection recognised as an occupational disease) in Germany between 01/01/2014 and 30/11/2016. The end points were results of monitorings carried out twelve weeks after the end of treatment (SVR12), side effects and the results of the assessment of reduced work ability after treatment. Multivariate logistic regression models were constructed to model SVR12.

**Results:**

The study population (*n* = 180) comprised 74% women, 90% of the participants had an HCV genotype 1 infection. Two-thirds had fibrosis or cirrhosis and were treatment experienced. The most common combined therapy was ledipasvir and sofosbuvir (49%). A DAA therapy with ribavirin was administered in 20% of cases, with (pegylated) interferon and ribavirin used in 2% of cases. The majority of therapies were completed without any side effects. The overall SVR12 rate was 94%. Significant independent predictor of decrease odds of SVR12 was liver cirrhosis. Positive effects on the healthcare personnel’s work ability were observed after successful therapy.

**Conclusion:**

High SVR12 rates were achieved in the sample population, with positive effects on their work ability. Early HCV therapy seems reasonable due to the increased chance of successful treatment of the infection.

**Electronic supplementary material:**

The online version of this article (10.1186/s12995-018-0197-6) contains supplementary material, which is available to authorized users.

## Background

The hepatitis C virus (HCV) is one of the most common infectious diseases in the world and develops into a chronic infection in up to 85% of cases. According to data from the World Health Organisation (WHO), approx. 71 million people globally have a chronic hepatitis C (CHC) infection [1, 2]. CHC is associated with higher morbidity and higher mortality [2, 3]. The use of direct-acting antiviral agents (DAAs) is currently leading to a major shift in the management of chronic HCV infections. DAAs open up the possibility for efficient oral therapy regimens with fewer side effects for patients with or without treatment experience. As recent studies have shown, interferon-free DAA treatments have achieved substantial sustained virological response rates (SVRs) of over 90% [4, 5]. For CHC infections, treatment success is achieved when the HCV virus’s RNA can no longer be detected in the blood (SVR), generally twelve weeks after the end of treatment [6, 7]. SVRs are associated with lowering morbidity and mortality caused by CHC [8, 9].

Healthcare personnel (HP) have contact with infected patients as part of their work. Injuries caused by sharp or pointed objects are some of the most commonly reported workplace accidents [10]. In Germany, reports detailing a reasonable suspicion of occupational illness are submitted to the statutory accident insurance carriers. Despite being on the decrease, Hepatitis C is still one of the most common infections leading to the recognition of an occupational illness among German HP. It is also the most common reason for newly approved retirement on the grounds of occupational disease [11]. Data analysis of an accident insurer shows that despite the number of cases has declined, the costs of HCV infection as an occupational disease (OD) have been significantly increasing over the past 15 years. These costs are the result of the increase in compensation payments for retirement on the grounds of OD and, since 2012, as a result of an increase in the cost of the drugs used to treat the infection [12]. The high costs of DAA therapies are offset by the potentially considerable benefits. The aim of this study is to investigate the treatment results of the DAA therapies in HP.

## Methods

Analysis of the DAA therapies using data from the Statutory Accident Insurance of the Health and Welfare Service (Berufsgenossenschaft für Gesundheitsdienst und Wohlfahrtspflege, BGW). This analysis was carried out in line with the Consensus German Reporting Standard for Secondary Data Analyses (STROSA) [13]. When applying for DAA therapy for a HP with an occupational CHC infection, the BGW collects data (using a standardised input mask (Excel table)) about the course of treatment for the purposes of conducting an analysis. For quality assurance purposes, this process was discussed and aided by the BGW’s Occupational Diseases working group. Following data was anonymised extracted from doctors’ letters and test results in the insured party’s medical files: genotype, reduced work ability (RWA) grading, treatments used, treatment status (naive/experienced), cirrhosis (yes/no), start and end of treatment (duration), treatment result (RNA evidence), side effects, and assessment of the RWA after DAA therapy. The analysis included data from insured individuals who completed DAA therapy between 01/01/2014 and 30/11/2016 and for whom treatment results were available twelve weeks after the end of therapy. In cases of treatment failure directly following the end of therapy, if there was no data available at twelve weeks after the end of treatment, it was also assumed that treatment had failed. Evidence of virus RNA twelve weeks after the end of treatment following previous ETR (end-of-treatment sustained virological response) was considered a relapse. The end points investigated were treatment success twelve weeks after the end of treatment (SVR12), side effects and the results of assessment of the RWA after the conclusion of DAA therapy.

### Determination of RWA in occupational disease procedures

Statutory accident insurance is one of the pillars of German social insurance. On the basis of the 7th German Social Security Code (Siebtes Buch, Sozialgesetzbuch, SGB VII), every employer is legally obliged to insure employees against accidents at work. The sponsorship of statutory accident insurance, the group of insured persons and the procedure in the event of a claim as well as the benefits in the event of an insured event are regulated in this code of law. The essential benefit for insured persons who get injured at the workplace following an accident or who suffer an occupational disease is the entitlement to compensation if their performance and thus their ability to work cannot be fully restored. This entitlement for the injured person’s pension is dependent on the assessment of the RWA and the extent to which the reduction in the physical and mental capacity of an insured person restricts their work opportunities. In the event of a complete loss of working ability (100%), a full pension is paid, which amounts to two thirds of the annual earnings before the occupational disease. In the case of a partial RWA, a partial pension corresponding to the degree of RWA is paid. The entitlement starts with a RWA of at least 20%. For claimants suffering HCV, the RWA is determined by the fibrosis stage and the degree of inflammatory activity of the disease. The initial assessment requires a detailed anamnesis, the clinical status and an abdominal sonography as well as a sufficient laboratory program with clinical-chemical, virological and immunoserological parameters. A reliable differential diagnostic and functional assessment of the liver situation needs to be ensured. Further assessment criteria are the probable duration and the course of the disease and the associated statement on the prognosis. A RWA of 20% should be granted when the CHC infection is accompanied with an increased concentration of the liver enzymes but not with a fibrosis of the liver, whereas a RWA of 50 to 100% should be granted when a cirrhosis is present. However, the grading is performed individually and personal circumstances like fatigue or depression should also be considered. The grading is performed by the case manager of the insurance with the support of a physician. The RWA determines the amount of the pension to be paid, even over the period of working life.

### Statistical analysis

Univariate comparisons were made to describe the correlations between treatment success (SVR12) and the categorical variables treatment status (naive/experienced), cirrhosis (yes/no), RWA (≤50%/> 50%) and gender. The significance of the correlations was tested using the Fisher’s exact test. Pearson’s correlation coefficient was calculated to measure the statistical association between age and RWA. Multivariate logistic regression models were constructed to model SVR12. Models included cirrhosis, prior treatment, gender, and age (as a continuous variable). Nagelkerke’s R-squared was calculated and used to derive Cohen’s effect size [14]. Using the measure according to Cohen, an *f*-value of 0.10 equates to a small effect, an *f*-value of 0.25 to a medium effect and an *f*-value of 0.40 to a large effect. For all comparisons, a *p*-Value < 0.05 was considered statistically significant. Data analysis was done using IBM SPSS Statistics version 23.

## Results

During the analysis period, a DAA therapy was administered to 180 HP and the SVR was determined twelve weeks after the end of treatment.

### Description of the sample (Table 1)

The mean age of the insured individuals was 62 years. For the period under analysis, 60% were under the statutory retirement age of 65 years. The sample population comprised 74% women. The most common type of infection among the sample was HCV genotype 1 (91%), with individual cases caused by viruses of genotypes 2, 3 or 4. No co-morbidities with Hepatitis B or HIV infections were documented. There were histological indications in 76% of the participants (fibrosis 43%; compensated or decompensated cirrhosis 24 and 9%, respectively) before the DAA therapy started and 72% had already experienced a prior treatment. More than 90% of the insured individuals had a RWA, approximately a third had a RWA of more than 50%.

**Table 1:** Baseline characteristics (*n* = 180)

Characteristic (missing values n/%)	Valid values	
	n	%
Gender		
Women	133	74
Men	47	26
Age		
Mean (SD)	62 (10)	
Median/Minimum/Maximum	62/21/88	
Age group		
30–39	4	2
40–49	12	7
50–59	50	28
60–64	42	23
≥ 65^a^	72	40
Genotype (11/6)		
1 sub-type not recognised	15	9
1a	50	29
1b	88	52
2	6	4
3	7	4
3a	2	1
4	1	< 1
RWA % (5/3)		
0 < 20	12	7
20	59	34
30–40	46	26
50–60	35	20
70–80	11	6
90–100	12	7
Stage of liver disease (28/16)		
No findings	37	24
Fibrosis	65	43
Compensated cirrhosis	37	24
Decompensated cirrhosis	13	9
Therapy status (21/12)		
Naive	45	28
Experienced	114	72

^a^statutory retirement age; *RWA* reduced work ability, *SD* standard deviation

### DAA-regimens and side effects (Table 2)

The most common treatment was a combination therapy of ledipasvir (LDV) and sofosbuvir (SOF) (*n* = 88/49%). A DAA therapy with ribavirin (RBV) was administered in 36 cases (20%), and (pegylated) interferon (PEG-IFN) and RBV were used in four cases (2%). Treatment duration was between eight and 24 weeks, with the majority lasting twelve weeks (71%). For 67% of insured individuals, the treatment was not associated with any side effects. The most common side effect was a combination of mild symptoms such as headaches, nausea and sleep disorders and/or fatigue (26%). 4% of the sample population suffered from skin reactions (pruritus to generalised skin rash, phototoxic reactions), and there were individual cases of decrease in haemoglobin, feelings of anxiety or depression and isolated gastrointestinal disorders. Side effects not linked to the DAA therapy, but seen as symptoms of an advanced CHC infection were summarised as other side effects (bleeding of the oesophageal varices, incipient hepatorenal syndrome). The observed side effects of a decrease in haemoglobin (*n* = 2) occurred in DAA therapy (LDV/SOF) combined with RBV in priory treated patients with liver cirrhosis. These therapies were conducted for twelve or 24 weeks and led to SVR12 in the insured individuals. Anxiety and depression were observed in patients with treatment experience and without liver cirrhosis (*n* = 2) receiving DAA therapies in combination with ribavirin or PEG-IFN (SOF/RBV/PEG-IFN; dasabuvir (DSV)/ombitasvir (OBV)/paritaprevir (PTV)/ritonavir (RTV)/RBV). These were completed after the standard twelve weeks and led to SVR12 in the insured individuals.

**Table 2:** DAA treatment regimens and results (*n* = 180)

Characteristic (missing values n/%)	Valid values	
	n	%
Therapies total (2/1)	178	
LDV, SOF	88	49
SOF, DCV	29	16
LDV, SOF, RBV	17	10
SOF, SMV	11	6
DSV, OBV, PTV, RTV, RBV	11	6
DSV, OBV, PTV, RTV	9	5
SOF, RBV	7	4
SOF, RBV, PEG-IFN	3	2
DCV, SOF, RBV	1	< 1
SOF, SMV, RBV	1	< 1
PEG-IFN, TVR, RBV	1	< 1
Therapy result		
Directly after therapy (1/< 1)	179	
ETR	173	97
Remission	6	3
Twelve weeks after therapy	180	
SVR12	170	94
Remission/viral load unchanged	6	> 2
Relapse	4	> 3
Side effects (13/7)	167	
None	110	66
Headaches, nausea, sleep disorder	43	26
Skin reactions	6	4
Anaemia	2	> 1
Depression, anxiety	2	> 1
Gastrointestinal disorders	2	> 1
Other^a^	2	> 1

^a^Manifestation of advanced infection (hepatorenal syndrome *n* = 1, bleeding of oesophageal varices n = 1); *DAA* direct-acting antiviral agents, *LDV* ledipasvir, *SOF* sofosbuvir, *DCV* daclatasvir, *RBV* ribavirin, *RTV* ritonavir, *SMV* simeprevir, *DSV* dasabuvir, *OBV* ombitasvir, *PTV* paritaprevir, *PEG-IFN* pegylated interferon, *TVR* telaprevir, *ETR* end-of-treatment response, *SVR12* sustained virologic response twelve weeks after therapy

### Treatment results and summary statistics

The ETR rate was 97% and the SVR12 rate was 94% (Table 2). Virus RNA could still be detected in six individuals directly after the end of treatment. At the results monitoring twelve weeks after the end of treatment, six patients showed no change or a reduction in viral load, while four patients had a relapse (Additional file 1: Table S1). The results of univariate and logistic regression analysis regarding treatment response (SVR12) are described in Table 3. Prior experience of treatment resulted in no significant difference in terms of SVR12 (naive *n* = 45/experienced *n* = 114: SVR12 98% vs 94%, *p* = 0.44), while there was a statistically significant difference with regard to cirrhosis status. For individuals without cirrhosis (*n* = 102), the treatment was statistically significant more likely to be successful than for patients with diagnosed cirrhosis (*n* = 50) (SVR12 98% vs 86%, *p* = 0.006). For insured individuals with a RWA grading of ≤50% (*n* = 139), the DAA treatment was also statistically significant more likely to be successful than for patients with a higher RWA grading (*n* = 36) (SVR12 97% vs 86%, *p* = 0.019). It was somewhat more likely for women (*n* = 133) to reach SVR12 after therapy than men (*n* = 47) (SVR12 96% vs 89%, *p* = 0.130). The multivariate regression showed that cirrhosis was a statistically significant independent predictor of decrease odds of SVR12. Participants with cirrhosis were 10 percentage points less likely to reach SVR12 in comparison to patients without cirrhosis. This resulted in an adjusted OR of 0.098 (95% CI 0.01–0.75; *p* = 0.03). With an increase in age, there was also an increase in the probability of achieving SVR12 (OR 1.11; 95% CI 1.01–1.23, *p* = 0.04). The variables of prior treatment (OR 0.42; 95% CI 0.05–3.92; *p* = 0.42) and gender (OR 0.41; 95% CI 0.08–2.18; *p* = 0.3) showed no statistically significant correlation with the end-point SVR12 in the multivariate analysis. Nagelkerke’s R-squared was 0.24, which corresponds to an f-value of 0.56, which Cohen classifies as a large effect size. The liver enzyme laboratory values (GOT, GPT, ɣGT) for 102 insured individuals at twelve weeks after the end of treatment were available at the time of the evaluation. For 90 (88%) of the individuals, the liver enzymes were in the normal range twelve weeks after the DAA therapy.

**Table 3:** Summary of univariate and logistic regression analysis for variables regarding treatment response SVR12 (*n* = 180)

Univariate analysis					
Variable	Missing values	n total	% SVR12 rates	OR (95% CI)	*p*-value
Cirrhosis (no/yes)	28	152 (102/50)	98% versus 86%	0.12 (0.03–0.62)	< 0.01
Treatment (naive/experienced)	21	159 (45/114)	97.8% versus 93.9%	0.35 (0.04–2.90)	NS
RWA (≤50%/> 50%)	5	175 (139/36)	97.1% versus 86.1%	0.18 (0.05–0.72)	< 0.05
Gender (women/men)	0	180 (133/47)	96.2% versus 87.5%	0.32 (0.09–1.19)	NS
Multivariate logistical regression model with SVR12 as end point					
Variable	Missing values	n total	OR (95% CI)	*p*-value	R^2^
Cirrhosis (no/yes)	34	146 (98/48)	0.098 (0.01–0.75)	< 0.05	0.242
Treatment (naive/experienced)	34	146 (42/104)	0.42 (0.05–3.92)	NS	
Gender (women/men)	34	146 (112/34)	0.41 (0.08–2.18)	NS	
Age^a^	34	146	1.11 (1.01–1.23)	< 0.05	

*SVR12* sustained virologic response twelve weeks after therapy, *RWA* reduced work ability; Dependent variables - the first category is established as a reference;

^a^as a continuous variable; OR (odds ratio) for the primary end point of SVR12; *NS* non-significant

### Evaluation of RWA after DAA therapy

Evaluation of RWA after DAA therapy was done for 115 (64%) of the insured individuals, on average nine months after the end of treatment. The RWA was adjusted for 87 (76%) of the insured individuals. The RWA determined before treatment lapsed for 56 individuals, 25 individuals had a decrease in the RWA after the evaluation and six increased their RWA (Table 4). Reasons for an increased RWA included liver transplant after successful DAA therapy, bleeding of the oesophageal varices and incipient hepatorenal syndrome. The correlation between age and RWA was low (*r* = 0.16, *p* = 0.04).

**Table 4:** Reduced work ability before and after DAA therapy (*n* = 115)

RWA as %		After DAA therapy						Overall	
Before DAA therapy		0	20	30–40	50–60	70–80	90–100	n	%
	0	6	0	0	0	0	0	6	5.2
	20	38	7	1	0	0	0	46	40
	30–40	15	4	7	1	0	0	27	23.5
	50–60	2	2	10	6	0	0	20	17.4
	70–80	1	0	1	0	4	3	9	7.8
	90–100	0	0	0	0	4	3	7	6.1
Overall	n	62	13	19	7	8	6	115	
	%	53.9	11.3	16.5	6.1	7	5.2	100	100

*RWA* reduced work ability, *DAA* direct-acting antiviral agents

## Discussion

Patients with and without experience of treatment who have a CHC infection achieved high SVR12 (94%) rates in the observed sample. Cirrhosis status (OR 0.098; 95% CI 0.01–0.75; *p* = 0.03) and age (OR 1.11; 95% CI 1.01–1.23; *p* = 0.04) had a significant correlation with treatment success. Significant independent predictor of decrease odds of SVR12 was liver cirrhosis. Even though, the correlation between age and SVR12 was statistically significant, advanced age is no barrier to DAA therapy. SVRs are associated with reducing morbidity and mortality resulting from a CHC infection, irrespective of individual cirrhosis status. They are also associated with an improvement in health-related quality of life [8, 9, 15, 16]. In the study population, positive effects on the patients’ RWA were observed on average nine months after successful completion of therapy. An evaluation was carried out in 64% of the insured individuals after DAA therapy, showing an improvement in work ability for more than 70% of those being analysed. Hence we assume that pension payments will decrease as well.

In a study with patients with a genotype 1 HCV infection, Backus et al. [17] investigated predictors of achieving SVR (at least ten weeks after the end of treatment) to determine the efficacy of LDV/SOF ± RBV and OBV/PTV/RTV/DSV ± RBV. The variables included in the multivariate analysis were treatment status (naive/experienced), ethnic background, body mass index (BMI), cirrhosis status (FIB-4 > 3.25), age and gender. SVR rates of 94% were achieved in the patient collective (average age: 61, 96% male, 23% with experience of treatment, 30% with cirrhosis). Cirrhosis status (OR 0.60; 95% CI 0–49–0.72, *p* = 0.001), having an African-American background (OR 0.71; 95% CI 0.59–0.86, p = 0.001) and a BMI of ≥30 kg/m^2^ (OR 0.73, 95% CI 0.60–0.89, *p* = 0.002) had a significant correlation with achieving SVR. In another study, no significant differences were found between treatment naive patients with and without cirrhosis in terms of SVR12 (97.9% vs 96.2%) [18]. Overall, the authors reported that no relapses were observed in patients once they had achieved SVR12. According to Zeuzem [19], the lack of evidence of HCV RNA twelve weeks after the end of DAA therapy signifies a permanent eradication of the virus. Relapses after this point are rare and generally take the form of a reinfection [19]. The most common DAA combination therapy administered in the observed sample was LDV/SOF (49%) and, according to Zimmermann et al. [20], was also the most commonly administered among patients with statutory health insurance in Germany (64%) in 2015. The frequency of the combination of DAA treatments with RBV was not quantified in that particular study. The authors stated a decrease in prescriptions of monthly PEG-IFN therapy regimes from around 2700 in January 2014 to around 650 in December 2015. In this study, a DAA therapy with RBV was administered in 36 cases (20%), and the combination with PEG-IFN and RBV was used in four cases (2%). Treatment was generally administered for twelve weeks (67%). Comparable treatment periods were also observed in the German Hepatitis Cohort (GECCO) [20]. The most commonly described side effects in our study, which were mainly described as mild, were nausea, headaches and sleep disorders, and were also listed by Zeuzem [19] for therapies with SOF and simeprevir (SMV). According to the author, photosensitivity reactions have also been observed during treatment using SMV. These were also observed in our study during combination treatment with SMV, without any consequences for the course of treatment. Decreased haemoglobin was observed in two DAA treatments with LDV/SOF in combination with RBV. The occurrence of haemolytic anaemia during treatment with RBV has been documented in the literature. The therapies were administered successfully (SVR12). In addition, side effects such as anxiety and depression were observed in two individuals with DAA treatments in combination with RBV or PEG-IFN (SOF/RBV/PEG-IFN, DSV/OBV/PTV/RTV/RBV). The treatments were successfully completed (SVR12). The occurrence of depression has been described in the literature both for PEG-IFN and RBV, and particularly when administered as a combination therapy [21, 22].

Cost effectiveness models analysed in the review by Nuno Solinis et al. [15] show that interferon-free DAA therapies are more cost effective than previous interferon-based therapies. The models also showed that early treatment is more cost-effective than therapy in later stages of the disease.

Data analyses showed an increase of costs associated with HCV as an OD in the period from 2000 to 2014 [12]. Despite lower OD HCV prevalence, the cost related to occupational exposure have increased in this period. About of € 87.9 million were spend by the statutory accident insurance for HCV as OD (*n* = 1.121), of which 60% were attributable to pension payments and around 15% to expenses for pharmaceuticals and other medicines. The cost of CHC are largely defined by the rising expenses for pensions due to increase in RWA. However, there was a strong rise in cost of drug therapy in 2012 to 2014 from € 1.7 to € 2.7 million. In 2015, the cost of antiviral drugs for CHC as OD increased to approximately € 11.9 million [23]. As reported by Zimmermann et al. [20], in 2014 about € 664 million were spent on HCV antivirals by the German statutory health insurance (SHI) and approximately € 1.3 billion in 2015. In Germany, more than 70 million persons are insured by the SHI, which represents about 85% of the German population. DAAs initially involve higher expenses, but as an effective treatment they may reduce long term treatment costs. Although the therapy success is convincing, and in Germany anyone with CHC has general access to DAA therapy, prescriptions have been showing a downward trend among patients with statutory health insurance since the end of 2015 [20]. Some possible reasons listed are insecurities regarding the authorisation of treatments and a lack of clarity in the reimbursement system. As a result of the unspecific course of the disease, researchers assume that around 100,000 people in Germany may have an HCV infection and not know about it [24, 25]. According to the WHO, less than 5% of people with chronic hepatitis worldwide (hepatitis B and C) are aware of their status [26]. The lack of compulsory screening strategies for at-risk groups (e.g. HP, intravenous drug users (IVD), men who have sex with men (MSM) and migrants from countries with high prevalence rates) has been criticised internationally [21, 25, 26]. The implementation of screening strategies to identify infected individuals and to interrupt the channels of infection is a major step in the prevention of the disease [24, 25]. There is no vaccine against HCV infection and a successfully treated infection does not offer protection against reinfection [19, 27]. To prevent reinfections (i.e. from needle-stick injuries) it is advisable to include employees with DAA therapy into regular check-up schemes.

The case figures presented here do not provide a complete picture for occupational HCV infections. The BGW only records notifications of occupational illness from employees of non-government institutions. This evaluation is based on register data with specific sociodemographic information, the data is not clinical in nature and not always complete (e.g. missing’s in stage of liver disease (15.6%) and treatment experience (11.7%)). Information about co-infections with HBV or HIV could not be evaluated from this database in a statistically valid way because it has not been requested in a standardised form. However, we assume that there is a lower likelihood of co-infection with HBV or HIV because the individuals in the study were HP. These employees have regular check-ups from occupational healthcare practitioners, which include checking their HBV vaccination status. Three quarters of the sample size in the study are female. Men are more commonly affected by HCV infections than women. Men are more often in at-risk groups, such as the IVD and MSM groups, and are more often co-infected with HIV [25, 26]. Interim results from the GECCO study confirm that men are significantly more likely to have an HCV/HIV co-infection than women [4].

Only the occupational acquired HCV as insured event was taken into consideration in this study. We do not have information on co-morbidities not related to the CHC infection. If the working ability is reduced by several insured events, the RWA is determined separately for each insured event. As these co-morbidities are not considered when grading the RWA for CHC the confounding effect should be minor. We expected that with increasing age the RWA would also rise. However, we observed only a low positive correlation between age and RWA (*r* = 0.16, *p* = 0.03).

Our report is based on the experience of a major German statutory accident insurance with the treatment of Hep C carriers in HP with new antiviral drugs. It is a great achievement that HP with a CHC and failed treatments in the past can be successfully treated. The major outcome is the high therapeutic success (SVR12 94%), although the majority of insures had liver disease. Even if not all of them are still in employment and actively working as HP, a decrease in RWA signifies a reduction in individual disease burden and in pension’s payments.

## Conclusions

Patients with and without experience of treatment who have a CHC infection achieved high SVR rates in the observed sample. Early treatment is preferable in order to keep the individual disease burden as low as possible. It appears that early treatment is also indicated for cost reasons, e.g. as a result of the influence of cirrhosis on treatment success. DAA therapies make it possible to eradicate the HCV viruses, thus enabling early recovery from the chronic infection. SVRs are associated with substantial reductions in the individual disease burden and with the patient maintaining their ability to work. Over the long term, this will probably lead to cost savings for statutory accident insurers and also for other social security systems. However, we require long-term experiences with DAA therapies in order to be able to reliably interpret the results. Successful completion of treatment does not provide protection from reinfection. HCV screening programmes for at-risk groups are therefore essential, even after successful treatment.

## Additional file

Additional file 1: **Table S1.** Characteristics according to treatment response without ETR and/or SVR12. (DOCX 14 kb)

## Abbreviations

BGW: Berufsgenossenschaft für Gesundheitsdienst und Wohlfahrtspflege
CHC: Chronic hepatitis C
DAA: Direct-acting antiviral agent
DSV: Dasabuvir
ETR: End-of-treatment sustained virological response rate
GECCO: German Hepatitis Cohort
HBV: Viral hepatitis B
HCV: Viral hepatitis C
HIV: Human immunodeficiency virus
HP: Healthcare personnel
IVD: Intravenous drug users
LDV: Ledipasvir
MSM: Men who have sex with men
OBV: Ombitasvir
OD: Occupational disease
PEG-IFN: Pegylated interferon
PTV: Paritaprevir
RBV: Ribavirin
RNA: Ribonucleic acid
RTV: Ritonavir
RWA: Reduced work ability
SOF: Sofosbuvir
STROSA: A Consensus German Reporting Standard for Secondary Data Analyses
SVR: Sustained virological response rate
WHO: World Health Organisation

## References

[1] Askarian M, Yadollahi M, Kuochak F, Danaei M, Vakili V, Momeni M (2011). Precautions for health care workers to avoid hepatitis B and C virus infection. The international journal of occupational and environmental medicine.

[2] Global Hepatitis Report 2017. Geneva: World Health Organization; 2017. Licence: CC BY-NC-SA 3.0 IGO. http://www.who.int/hepatitis/publications/global-hepatitis-report2017/en/. Accessed 15 Sept 2017.

[3] Sarrazin C, Berg T, Buggisch P, Dollinger MM, Hinrichsen H, Hofer DH (2015). Aktuelle Empfehlung zur Therapie der chronischen Hepatitis C S3 guideline hepatitis C addendum. Z Gastroenterol.

[4] Ingiliz P, Christensen S, Kimhofer T, Hueppe D, Lutz T, Schewe K, Busch H, Schmutz G, Wehmeyer MH, Boesecke C (2016). Sofosbuvir and Ledipasvir for 8 weeks for the treatment of chronic hepatitis C virus (HCV) infection in HCV-Monoinfected and HIV-HCV-Coinfected individuals: results from the German hepatitis C cohort (GECCO-01). Clinical infectious diseases: an official publication of the Infectious Diseases Society of America.

[5] Lubel J, Strasser S, Stuart KA, Dore G, Thompson A, Pianko S et al. Real-world efficacy and safety of ritonavir-boosted paritaprevir, ombitasvir, dasabuvir +/- ribavirin for hepatitis C genotype 1 - final results of the REV1TAL study. Antivir Ther. 2017;22(8):699–710. 10.3851/IMP3168. 10.3851/IMP3168 28422043

[6] Sarrazin C, Berg T, Ross RS, Schirmacher P, Wedemeyer H, Neumann U (2010). Prophylaxis, diagnosis and therapy of hepatitis C virus (HCV) infection: the German guidelines on the management of HCV infection. Zeitschrift fur Gastroenterologie.

[7] Westbrook RH, Dusheiko G (2014). Natural history of hepatitis C. J Hepatol.

[8] Gonzalez-Grande R, Jimenez-Perez M, Gonzalez Arjona C, Mostazo TJ (2016). New approaches in the treatment of hepatitis C. World Journal of Gastroenterology : WJG.

[9] Tada T, Kumada T, Toyoda H, Kiriyama S, Tanikawa M, Hisanaga Y et al. Viral eradication reduces all-cause mortality in patients with chronic hepatitis C virus infection: a propensity score analysis. Liver Int. 2016;36:817–26. 10.1111/liv.13071. 10.1111/liv.13071 26787002

[10] Nienhaus A, Kesavachandran C, Wendeler D, Haamann F, Dulon M (2012). Infectious diseases in healthcare workers - an analysis of the standardised data set of a German compensation board. J Occup Med Toxicol..

[11] Dulon ML, Lisiak B, Wendeler D, Nienhaus A, Occupational infectious diseases in healthcare workers (2014). Data from the institution for statutory accident insurance and prevention in the health and welfare services. Zbl Arbeitsmed.

[12] Westermann C, Dulon M, Wendeler D, Nienhaus A (2016). Hepatitis C among healthcare personnel: secondary data analyses of costs and trends for hepatitis C infections with occupational causes. J Occup Med Toxicol.

[13] Swart E, Bitzer EM, Gothe H, Harling M, Hoffmann F, Horenkamp-Sonntag D et al. [A Consensus German Reporting Standard for Secondary Data Analyses, Version 2 (STROSA-STandardisierte BerichtsROutine fur SekundardatenAnalysen)]. Gesundheitswesen (Bundesverband der Ärzte des Öffentlichen Gesundheitsdienstes (Germany)). 2016. 10.1055/s-0042-108647 27351686

[14] Cohen J, Cohen P, West SG, Aiken LS. Applied multiple regression/correlation analysis for the behavioral sciences (3rd ed.). Mahwah: Lawrence Erlbaum; 2003.

[15] Nuno Solinis R, Arratibel Ugarte P, Rojo A, Sanchez GY (2016). Value of treating all stages of chronic hepatitis C: a comprehensive review of clinical and economic evidence. Infectious diseases and therapy.

[16] Stahmeyer JT, Krauth C, Bert F, Pfeiffer-Vornkahl H, Alshuth U, Huppe D (2016). Costs and outcomes of treating chronic hepatitis C patients in routine care - results from a nationwide multicenter trial. J Viral Hepat.

[17] Backus LI, Belperio PS, Shahoumian TA, Loomis TP, Mole LA (2016). Comparative effectiveness of ledipasvir/sofosbuvir +/− ribavirin vs. ombitasvir/paritaprevir/ritonavir + dasabuvir +/− ribavirin in 6961 genotype 1 patients treated in routine medical practice. Aliment Pharmacol Ther.

[18] Lawitz E, Makara M, Akarca US, Thuluvath PJ, Preotescu LL, Varunok P (2015). Efficacy and safety of Ombitasvir, Paritaprevir, and ritonavir in an open-label study of patients with genotype 1b chronic hepatitis C virus infection with and without cirrhosis. Gastroenterology.

[19] Zeuzem S (2017). Treatment options in hepatitis C. Dtsch Arztebl Int.

[20] Zimmermann R, Kollan C, Ingiliz P, Mauss S, Schmidt D, Bremer V (2017). Real-world treatment for chronic hepatitis C infection in Germany: analyses from drug prescription data, 2010–2015. J Hepatol.

[21] Schäfer M, Schwaiger M (2003). Incidence, Pathoetiology and treatment of interferon-α induced neuro-psychiatric side Effetcs. Fortschr Neurol Psychiatr.

[22] Slim J, Afridi MS (2012). Managing adverse effects of interferon-alfa and ribavirin in combination therapy for HCV. Infect Dis Clin N Am.

[23] 14th Congress for Hospital Hygiene 2018, Berlin, March 18–21, 2018. Lecture on March 19 at 2.45 p.m.: [Vision 'zero infections' - can this be achieved in view of the temporal trend in occupational infections? A contribution to the discussion from the perspective of BGW - A. Nienhaus (Hamburg)]. https://www.congress-compact.de/pdf/2018_03_18-21_DGKH_Jahreskongress_Hauptprogramm.pdf. Accessed 10 Apr 2018.

[24] Ltd. LHP. THE ECO-HEP REPORT. A macroeconomic overview of viral hepatitis C in Germany https://www.leberhilfe-projekt.de/das-eco-hep-modell.html: Leberhilfe Projekt gUG (Liver Help Project Ltd.); Babette Herder and Achim Kautz; [2.8.2017]. Accssed 15 Sept 2017.

[25] Warpakowski A. Hepatitis C: Elimination in Europa möglich. Dtsch Ärztebl International.113(21):-20-.

[26] WHO. GLOBAL HEALTH SECTOR STRATEGY ON VIRAL HEPATITIS 2016–2021 [updated 2.8.2017; cited Juni 2016]. Available from: http://www.who.int/hepatitis/strategy2016-2021/ghss-hep/en/. Accessed 17 May 2018.

[27] Webster DP, Klenerman P, Dusheiko GM. Hepatitis C. Lancet 2015;385(9973):1124–1135. 10.1016/S0140-6736(14)62401-6 PMC4878852 25687730

