# Supplementary material for: Hepatitis C in healthcare personnel: secondary data analysis of therapies with direct-acting antiviral agents
Source: J Occup Med Toxicol. 2018 May 25;13:16. doi: 10.1186/s12995-018-0197-6 (PMC5970510; doi:10.1186/s12995-018-0197-6)
Supplement: Supplementary file 1 — Table S1. Characteristics according to treatment response without ETR and/or SVR12. (DOCX 14 kb) [file 12995_2018_197_MOESM1_ESM.docx]

**Additional file 1: Table S1. Characteristics according to treatment response without ETR and/or SVR12**

| Age | Gender | Genotype | Prior therapy | RWA | Cirrhosis | Therapy | Duration/  weeks | Side effect | ETR | SVR12 |
| --- | --- | --- | --- | --- | --- | --- | --- | --- | --- | --- |
| 46 | Female | 1b | Yes | 100 | Yes | LDV/SOF/RBV | 8 | Headaches/  nausea/sleep disorder | No | No ^a^ |
| 55 | Female | 1a | No | 40 | Yes | LDV/SOF/RBV | 12 | Headaches/  nausea/sleep disorder | Yes | Relapse |
| 60 | Female | 1 sub-type unknown | Yes | 60 | Yes | SOF/DSV | 24 | None | Yes | Relapse |
| 60 | Female | N/A | N/A | N/A | N/A | SOF/RBV | N/A | N/A | No | N/A |
| 64 | Female | 1b | Yes | 70 | Yes | SOF/SMV/RBV | 12 | None | Yes | Relapse |
| 81 | Female | 1b | N/A | 100 | Yes | LDV/SOF | 12 | None | Yes | Relapse |
| 55 | Male | 1a | Yes | 50 | No | DSV/OBV, PTV/RTV/RBV | 12 | N/A | No | No ^c^ |
| 57 | Male | 1b | Yes | 50 | No | LDV/SOF | 12 | None | No | No ^b^ |
| 60 | Male | 1 sub-type unknown | Yes | 60 | Yes | SOF/RBV | 24 | Headaches/  nausea/sleep disorder | No | N/A |
| 70 | Male | 1b | Yes | 50 | Yes | LDV/SOF/RBV | 12 | N/A | No | N/A |
| 77 | Male | 1b | Yes | 20 | N/A | LDV/SOF | 8 | None | No | Yes |

RWA, reduced work ability; ETR, end-of-treatment response; SVR12, sustained virologic response twelve weeks after therapy; LDV, ledipasvir; SOF, sofosbuvir; RBV, ribavirin; RTV, ritonavir; SMV, simeprevir; DSV, dasabuvir; OBV, ombitasvir; PTV, paritaprevir; a Assessment after 32 weeks (RWA 100%), repeated treatment offered; b Repeated treatment with SOF/SMV/RBV – discontinued after eight weeks due to non-compliance; c Repeated treatment: participation in POLARIS-1 study; N/A, not available
